# Ventricular Tachycardia Induced by Weight Loss Pills

**DOI:** 10.1155/2013/712383

**Published:** 2013-03-14

**Authors:** Manan Pareek, Nils Henrik Hansson, Erik Lerkevang Grove

**Affiliations:** Department of Cardiology, Aarhus University Hospital, Brendstrupgaardsvej 100, 8200 Aarhus, Denmark

## Abstract

A previously healthy 29-year-old man was admitted with palpitations, dizziness, and near-syncope after he had recently started taking weight loss pills purchased on the internet. The pills contained caffeine and ephedrine. An electrocardiogram and telemetry revealed multiple episodes of non-sustained monomorphic ventricular tachycardia, which was successfully treated with amiodarone. In conclusion, unauthorized weight loss pills can be harmful. In particular, ephedrine-containing drugs carry a risk of ventricular tachycardia and should be discouraged.

## 1. Introduction

Ventricular tachycardia (VT) is a potentially life-threatening arrhythmia that originates in one of the ventricles of the heart. VT can be classified into monomorphic and polymorphic rhythms. Monomorphic VT has the same QRS configuration from beat to beat, suggesting a stable origin of tachycardia from a focus or a structural substrate [1]. In the absence of structural heart disease, this arrhythmia most often originates from the ventricular outflow tracts and left ventricular fascicles; however, it may be idiopathic or drug induced [2, 3]. This paper describes a young man presenting with monomorphic VT after consumption of ephedrine- and caffeine-containing weight loss pills purchased on the internet.

## 2. Case Report

A previously healthy 29-year-old man with no family history of cardiovascular disease was admitted to the emergency room due to palpitations, dizziness, and near-syncope during weightlifting. Three days prior to admission, an episode of less severe but similar symptoms was reported. The patient appeared clinically stable on physical examination, and vital signs were reported as a blood pressure of 135/75 mmHg, pulse 121 beats per minute, oxygen saturation 94%, respiratory rate 20 per minute, and central body temperature of 37.4°C. 

**Figure 1:**
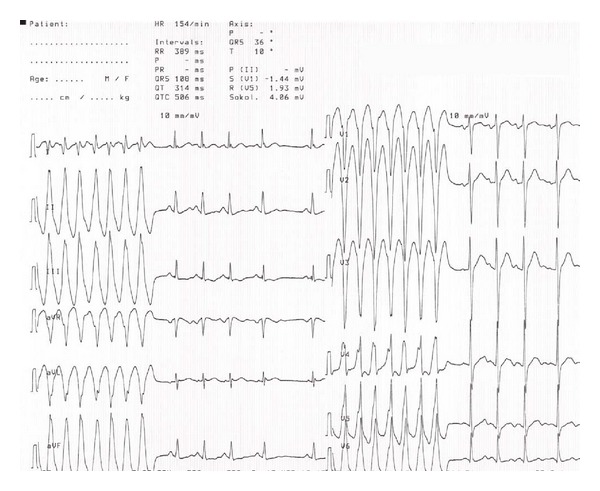
Electrocardiogram showed sinus tachycardia and non-sustained, monomorphic ventricular tachycardia.

**Figure 2:**
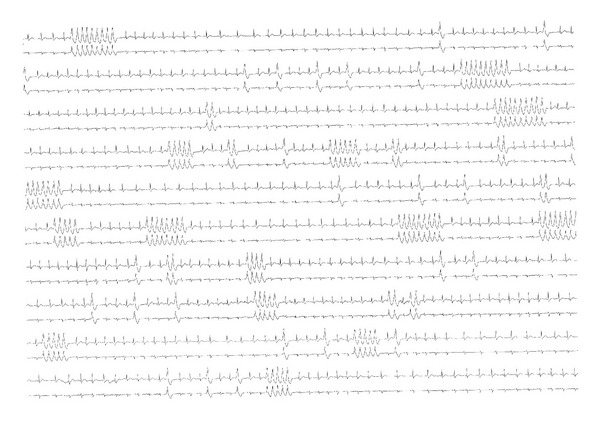
ECG telemetry with sinus tachycardia, ventricular extrasystoles, and multiple episodes of non-sustained monomorphic ventricular tachycardia.

The initial electrocardiogram (ECG) showed sinus tachycardia with a rate of 147 beats per minute and an episode of non-sustained, monomorphic VT. Consequently, the patient was transferred to the department of cardiology for ECG-monitoring and further evaluation. An ECG (Figure 1) confirmed the presence of sinus tachycardia and non-sustained VT, and an echocardiogram showed a structurally normal heart with a normal left ventricular ejection fraction. Initial blood tests, including electrolytes and high-sensitivity cardiac troponin T (hs-cTnT), were within the reference range. Due to multiple paroxysms of non-sustained monomorphic VT (Figure 2), the patient was given a 300 mg bolus of amiodarone intravenously over 20 minutes, which rapidly led to stabilization of the heart rhythm. The VT resolved completely within three hours. 

The patient did not take any prescription drugs. However, he was taking one capsule of weight loss pills daily, initiated one week prior to admission. He had purchased the product, which contains ephedrine, caffeine, and aspirin, on the internet. Since no other risk factors for VT were present, these capsules were the most likely cause of arrhythmia. 

The patient was monitored for another 24 hours without recurring episodes of VT. The corrected QT-interval (QTc) was normal during the entire admission, and hs-cTnT values remained within the reference range during serial measurements. The patient was discharged without further evaluation or follow-up.

## 3. Discussion

Ephedrine is a phenylalanine-derived alkaloid, which is extracted from the Chinese botanical ephedra (ma-huang). The compound is a mixed sympathomimetic agent; it enhances the release of noradrenaline from sympathetic neurons and exhibits direct alpha- and beta-adrenoceptor agonism [4, 5]. Ephedrine was once widely used in the treatment of asthma and nasal congestion. The use, however, rapidly declined, as the sympathomimetic effect was associated with adverse cardiovascular events, for example, acute myocardial infarction, severe hypertension, myocarditis, and stroke [4, 5]. Furthermore, cardiac refractory periods are shortened, permitting the development of potentially life-threatening re-entrant tachyarrhythmias [5].

 Ephedrine is present in a variety of dietary supplements claiming to improve athletic performance and to promote weight loss. Several of these products also contain caffeine, which may enhance the adverse cardiovascular effects of ephedrine by competitively antagonizing adenosine-receptors as well as augmenting endogenous catecholamine release. These mechanisms inhibit vasodilation and increase blood pressure, and the catecholamine release also leads to a general stimulation of the cardiovascular system [6, 7]. Ephedrine results in a modest but statistically significant weight loss, and caffeine is likely to enhance this effect. However, no effect on athletic performance has been documented [4, 8].

The manufacture and distribution of ephedra-containing dietary supplements were banned by the American Food and Drug Administration in 2004, due to the documented side effects of the substance, including an increment of blood pressure and imposed stress on the cardiovascular system [9]. However, several ephedrine-containing weight loss pills continue to be readily available online. Thus, this paper serves as a reminder of the potential hazards associated with ephedrine-containing products marketed as dietary supplements, as they may cause potentially life-threatening adverse cardiovascular events. 

## References

[1] John RM, Tedrow UB, Koplan BA (2012). Ventricular arrhythmias and sudden cardiac death. *The Lancet*.

[2] Prystowsky EN, Padanilam BJ, Joshi S, Fogel RI (2012). Ventricular arrhythmias in the absence of structural heart disease. *Journal of the American College of Cardiology*.

[3] Zipes DP, Camm AJ, Borggrefe M (2006). European Heart Rhythm Association; Heart Rhythm Society; American College of Cardiology; American Heart Association Task Force; European Society of Cardiology Committee for Practice Guidelines. ACC/AHA/ESC 2006 guidelines for management of patients with ventricular arrhythmias and the prevention of sudden cardiac death: a report of the American College of Cardiology/American Heart Association Task Force and the European Society of Cardiology Committee for Practice Guidelines (Writing Committee to Develop Guidelines for Management of Patients With Ventricular Arrhythmias and the Prevention of Sudden Cardiac Death). *Journal of the American College of Cardiology*.

[4] Dietary Supplement Fact Sheet Ephedra and Ephedrine Alkaloids for Weight Loss and Athletic Performance. http://ods.od.nih.gov/factsheets/EphedraandEphedrine-HealthProfessional/.

[5] Haller CA, Benowitz NL (2000). Adverse cardiovascular and central nervous system events associated with dietary supplements containing ephedra alkaloids. *New England Journal of Medicine*.

[6] Benowitz NL (1990). Clinical pharmacology of caffeine. *Annual Review of Medicine*.

[7] Robertson D, Froelich JC, Carr RK (1978). Effects of caffeine on plasma renin activity, catecholamines and blood pressure. *New England Journal of Medicine*.

[8] Shekelle PG, Hardy ML, Morton SC (2003). Efficacy and safety of ephedra and ephedrine for weight loss and athletic performance: a meta-analysis. *Journal of the American Medical Association*.

[9] FDA Acts to Remove Ephedra-Containing Dietary Supplements From Market. http://www.fda.gov/NewsEvents/Newsroom/PressAnnouncements/2004/ucm108379.htm.

